# Minimally Invasive 2D Navigation-Assisted Treatment of Thoracolumbar Spinal Fractures in East Africa: A Case Report

**DOI:** 10.7759/cureus.507

**Published:** 2016-02-23

**Authors:** Innocent Njoku, Othman Wanin, Anthony Assey, Hamisi Shabani, Japhet G Ngerageza, Connor D Berlin, Roger Härtl

**Affiliations:** 1 Department of Neurosurgery, Weill Cornell Medical College, New York Presbyterian Hospital, New York; 2 Department of Orthopedics, Muhimbili Orthopedic Institute (MOI), Dar es Salaam, Tanzania; 3 Department of Neurosurgery, Muhimbili Orthopedic Institute (MOI), Dar es Salaam, Tanzania

**Keywords:** neuronavigation, spinal trauma, global health, surgery as a global health issue, neurosurgery, neurotrauma, minimally invasive spine surgery (miss)

## Abstract

Spinal surgery under Eastern-African circumstances is technically demanding and associated with significant complications, such as blood loss, infection, and wound breakdown. We report a spinal trauma case that was performed using minimally invasive surgery (MIS) and navigation, and hypothesize that these newer techniques may enable surgeons to perform effective spinal surgery with minimal complications and good outcomes.

During the 2014 First Hands-on Neurotrauma Course held in Dar es Salaam, Tanzania, we successfully performed three minimally invasive and two-dimensional (2D) navigated spinal surgeries to decompress and stabilize patients with complete and incomplete spinal injuries. In this report, we present a case of a paraplegic patient with a T12 burst fracture who tolerated MIS surgery with no intraoperative complications, and is doing well with no postoperative complications one year after surgery.

Minimally invasive spinal surgery and 2D navigation may offer advantages in resource-poor countries. As part of the Weill Cornell Tanzania Neurosurgery project and in conjunction with the Foundation for International Education in Neurological Surgery (as well as other organizations), further experiences with 2D navigation and MIS surgery will be recorded in 2015. A neurotrauma registry has already been implemented to better understand the current management of neurotrauma in Eastern Africa.

## Introduction

Traumatic brain and spine injury is a leading cause of morbidity and mortality in young adults worldwide. We have recently shown that neurotrauma constitutes one of the most important neurosurgical problems in Tanzania [1]. Challenges in delivering surgical care, particularly in resource-limited environments, remains a major global health concern. The lack of trained surgeons, technological shortages, and overall poor infrastructure are contributing factors [2]. Current methods of treating spinal neurotrauma, especially in Eastern Africa, rely either on non-operative treatment or on invasive open procedures. Macrosurgical exposures are associated with adverse effects, including significant muscle damage, increased bleeding, neuromuscular denervation, reduced local blood supply, and a greater demand for pain medication [3-4]. Furthermore, complications, specific to resource-limited environments, also include higher rates of infection, wound breakdown, and prolonged bed immobility.

Minimally invasive surgery (MIS) with navigation increases the accuracy of pedicle screw placement in the thoracic and lumbar spine, and can enable surgeons to perform complex operations with fewer complications [5]. MIS surgery decreases postoperative pain, reduces infection rates, and overall morbidity [6]. In one recent example, Dr. Michael Wang successfully demonstrated the potential benefits of treating traumatic neurosurgical patients with spinal fractures in Haiti using minimally invasive, percutaneous stabilization surgery [6].

Despite the economic and infrastructural challenges in Eastern Africa, we explored the feasibility of utilizing more advanced technologies, such as MIS and neuronavigation. The 2D Kick^®^ system (BrainLab AG, Washington, IL) is a relatively inexpensive navigational system (compared to 3D navigation) that combines the benefits of navigation with affordable purchasing and maintenance costs; therefore, its feasibility and use can be translated to assist surgeries in resource-limited regions of the world.

To illustrate the practicality of this concept, we describe a patient who underwent an MIS procedure performed with 2D navigation to stabilize and decompress a thoracic burst fracture, in the setting of the Muhimbili Orthopedic and Neurosurgical Institute in Dar es Salaam, Tanzania. 

## Case presentation

**Figure 1:**
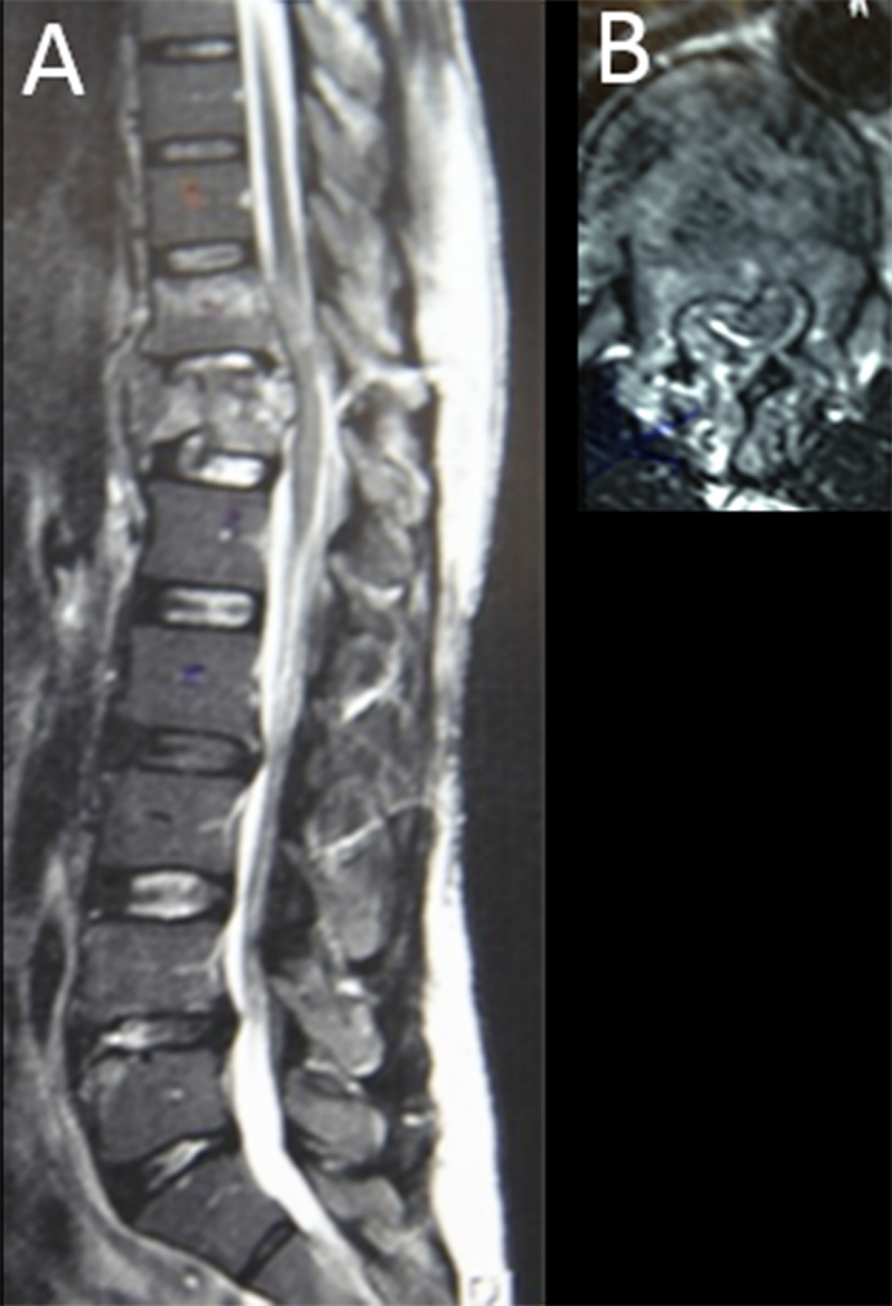
Burst Fracture at T12 (a) Sagittal and (b) axial MRI scan of the patient’s stable burst fracture at T12.

Three patients with spinal cord injuries were treated using 2D navigation and less invasive neurosurgical techniques. The Tanzanian surgeons performed the procedure while the visiting surgeons demonstrated and assisted. We present a 47-year-old man who sustained a T12 burst fracture after falling from a tree (Figure 1).

A thorough evaluation determined the patient had lost motor and sensory sensation in both lower limbs due to spinal cord injury, (ASIA grade A, American Spinal Injury Association schema) [7]. The decision was made to offer this patient focal decompression and fixation to stabilize the spine from T10 – L2. Informed patient consent was obtained, and the surgery was conducted ten days after the trauma.

**Figure 2:**
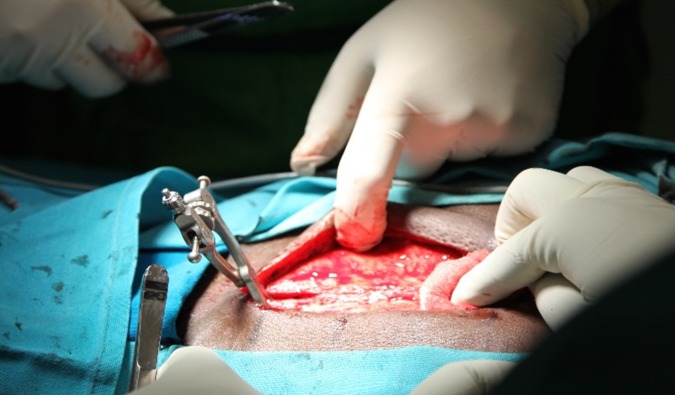
Interfascial Plane Intra-operative view of the midline incision demonstrating an interfascial plane.

The patient was intubated and anesthetized, placed carefully in a prone position on a standard operating room table, and prepped in standard fashion. A midline incision was made, and the muscle fascia was exposed from T10 to L2 via a subcutaneous plane (Figure 2). A reference array for the navigation system was placed on the L2 spinous process via a small, fascial midline incision.

**Figure 3:**
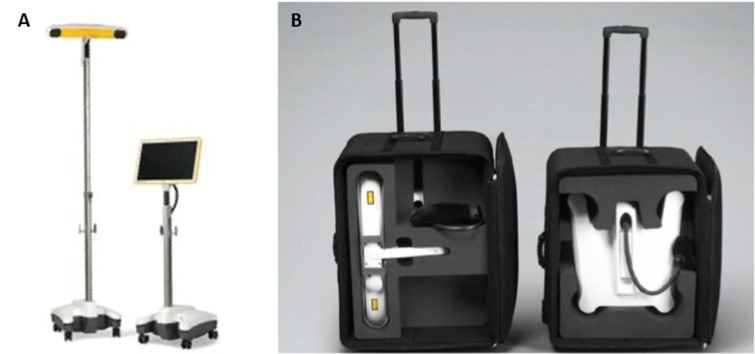
BrainLab Kick® 2D Navigation System (a) The BrainLab Kick® 2D navigation system disassembled and (b) assembled.

The BrainLab Kick® 2D portable navigation system with the Spine and Trauma 2D 3.1 software was used. AP and lateral fluoroscopy images were registered in reference to the patient using the Medtronic X-Stop*^PK^*^®^ device (Medtronic, Minneapolis, MN) (Figure 3). For the purpose of the week-long neurotrauma course and the surgeries, the Kick® navigation platform, which is compatible with most C-arms, the Depuy Synthes MATRIX™ minimally invasive pedicle screw system (Depuy Synthes Co., Switzerland), tubular retractors system with LifeSpine bayonetted instruments, and Anspach surgical drills were brought to Tanzania (Figure 3).

True AP and lateral fluoroscopy shots were obtained with a locally available Phillips BV Endura C-arm and loaded into the Kick® portable navigation platform. Multiple images were obtained and stored in the system for later use. Two lateral shots were taken for instrumentation of multiple spinal levels with the endplates and pedicles clearly visible and parallel. On AP view, the spinous processes were midline and the pedicles were symmetric. Multiple AP shots were taken in order to visualize the endplates and the pedicles clearly at each level. The accuracy of the navigation probe was confirmed by pointing to the tip of a transverse process through a small, fascial incision.

The ideal incision site for each pedicle trajectory was determined using the navigated pointer with the 'offset' function. One small 1.5 cm transverse fascial incision per screw was then made using a No. 15 blade. Transverse incisions are preferable to longitudinal incisions in order to avoid connection of individual longitudinal incisions, which can lead to muscle herniation and the inability to later close the fascial incision. Gentle, single-finger dissection was used to dissect the muscle and identify the correct entry point for the pedicle screw. In the thoracic spine, it may be helpful to use a small Kelly clamp to be able to penetrate and spread the muscle layers more easily and with minimal manipulation of the bony anatomy.

Drilling, tapping, and screw insertion was achieved using a navigated guide tube as previously described (part of the Depuy Synthes Matrix pedicle screw set) [8]. The advantage of this technique is that it eliminates the need of K-wires and greatly simplifies the navigation workflow since only one instrument (the guide tube) is navigated.

**Figure 4:**
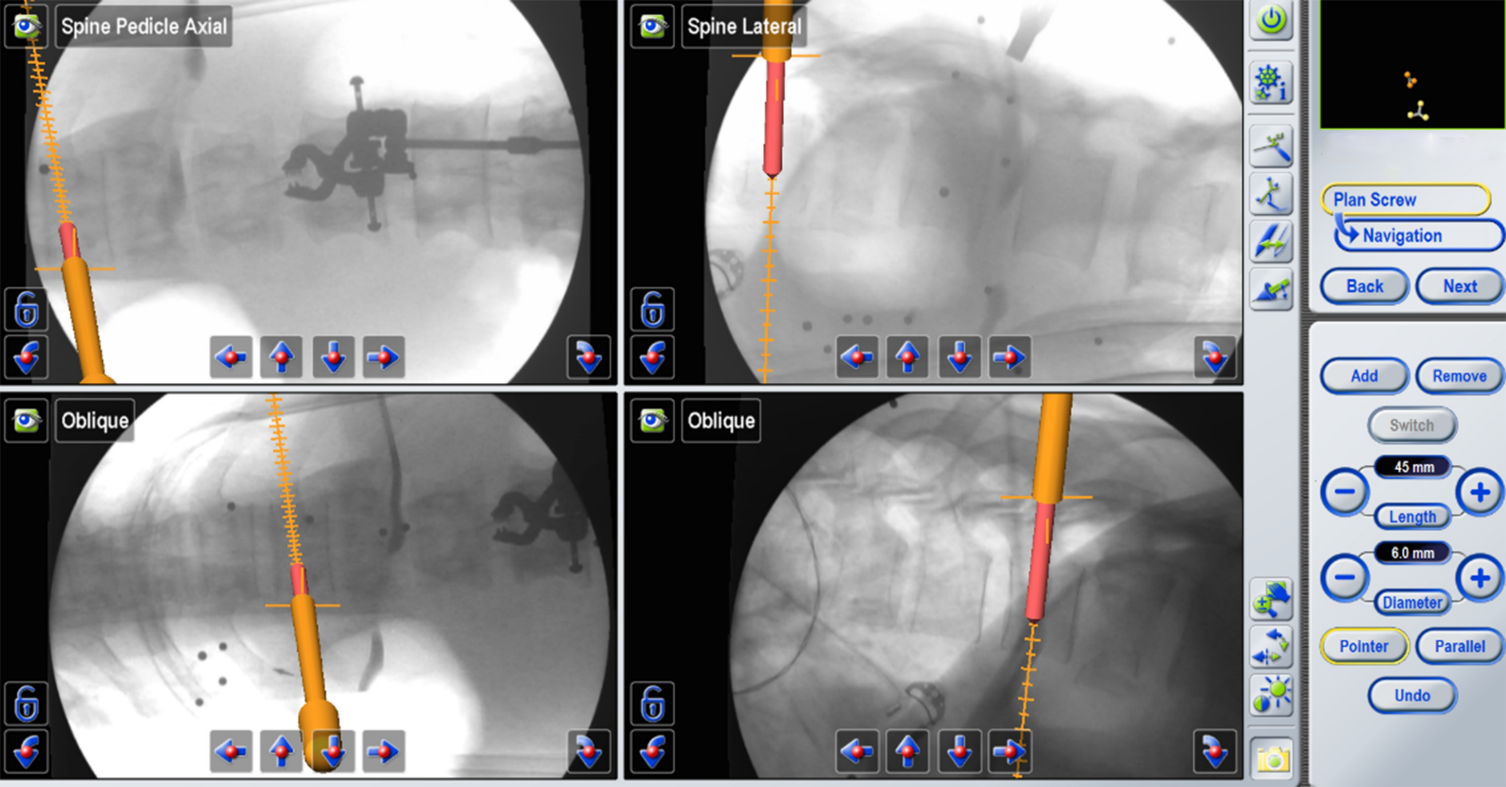
Ideal Pedicle Entry Point BrainLab simulation to determine the ideal pedicle entry point, trajectory, and the appropriate pedicle screw size.

The navigated guide tube attached to a Brainlab reference array was used to determine the ideal pedicle entry point and trajectory. An appropriate-sized pedicle screw was simulated using the navigation software (Figure 4).

**Figure 5:**
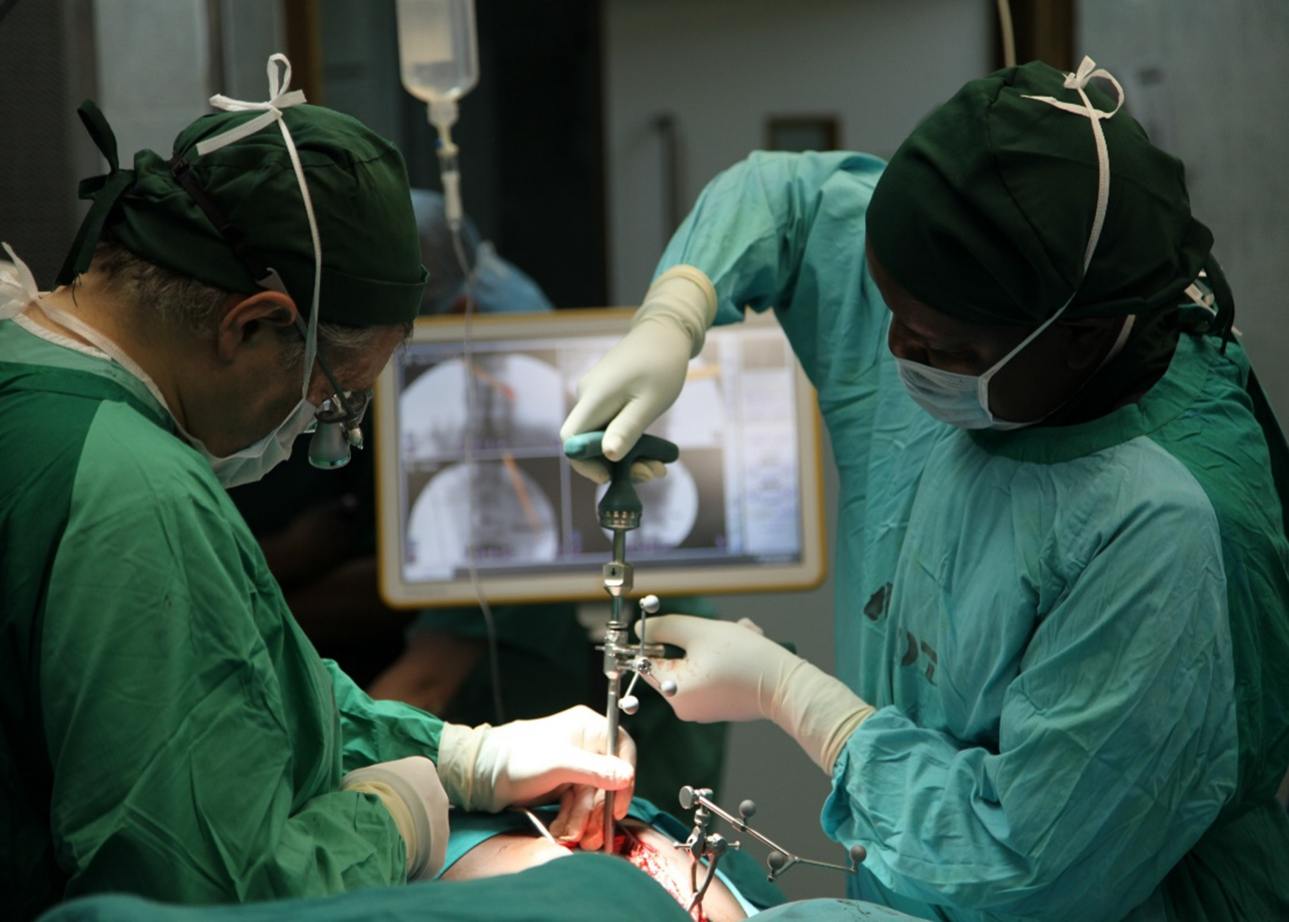
Intra-operative Screw Placement Intra-operative image demonstrating screw placement at the lateral midpoint of the pedicle.

The navigated guide tube was then gently impacted so that the teeth would hold on to the bony anatomy. A battery driven drill was then used, and a 35 mm hole was prepared (the drill and tap cannot advance deeper than 35 mm). The hole was then tapped, and a ball-tip instrument was used through the guide tube to detect potential breaches and to confirm the integrity of the pedicle hole. An appropriate pedicle screw without a screw head was inserted (Figure 5). We used a MATRIX^®^ (Depuy Synthes) system with modular screw heads.

**Figure 6:**
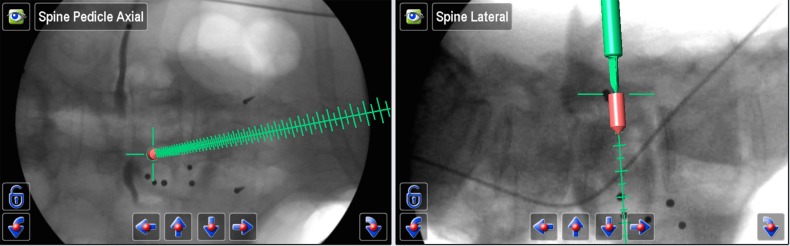
Ideal Medial/Lateral Trajectory BrainLab simulation to determine the ideal medial/lateral trajectory.

In order to determine the ideal medial/lateral trajectory and avoid medial pedicle violation, we find it helpful to initially simulate a pedicle screw that is no longer than the pedicle, typically about 1.5 cm (Figure 6). If this short, simulated screw does not breach the medial border of the pedicle on the AP view and seems to be in perfect medial/lateral position, we then lengthen the pedicle screw simulation until the desired screw length is achieved.

**Figure 7:**
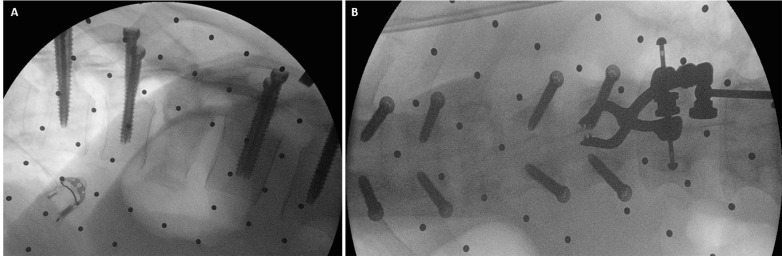
Intra-operative Fluoroscopy (a) Intra-operative AP and (b) lateral images demonstrating accurate screw placements

This simple technique avoids a medial violation of the pedicle wall that could result in potential nerve/spinal cord injury. After all of the screws were inserted, AP and lateral views were obtained to confirm the accurate placement of all screws (Figure 7).

**Figure 8:**
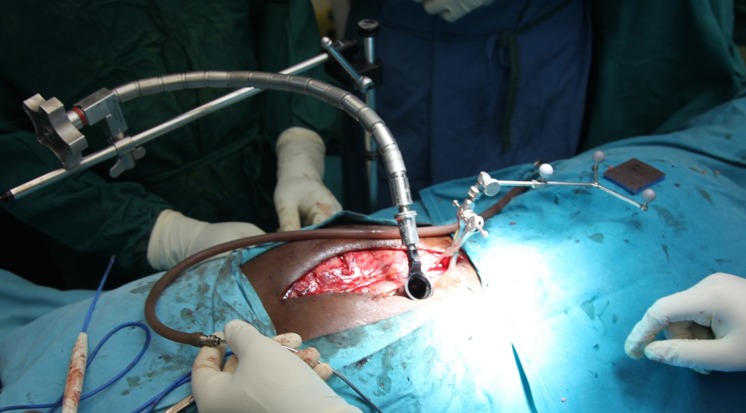
Tubular Positioning Intra-operative image showing tubular dilators and retractors.

Through a separate, fascial incision, approximately 1.5 cm lateral to the midline, a series of tubular dilators were placed at the T12 level and a 19 mm tubular retractor was placed for retraction (Figure 8). Under loupe magnification, headlight illumination, and using a high-speed Anspach hand drill, a T12 hemilaminectomy was performed and the ligamentum flavum was exposed. The spinous process and the contralateral lamina were undercut. Ligament and bone were carefully removed using small, Kerrison rongeurs and the spinal cord was decompressed bilaterally. Navigation was used at this point in order to confirm adequate bilateral pedicle-to-pedicle decompression from above the T12 pedicle down to L1.

**Figure 9:**
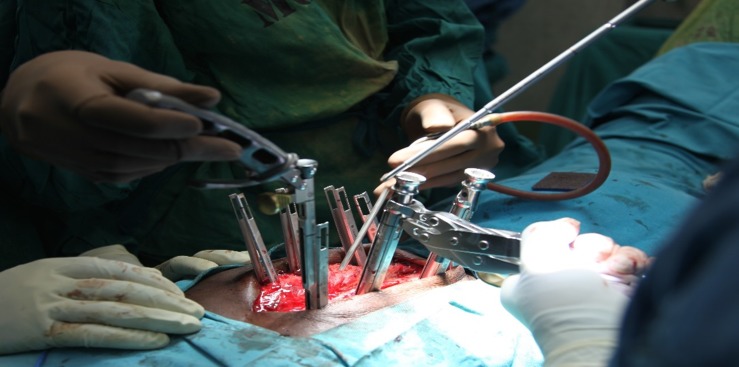
Rod Insertion Intra-operative view showing rod insertion through assembled screw towers.

Next, the screw heads with the extension sleeves were attached, and bilateral rods were placed through a subfascial corridor (Figure 9). We used a straight rod, and the rod length was approximated using navigation. The rod was then locked into place using caps and the towers were removed.

**Figure 10:**
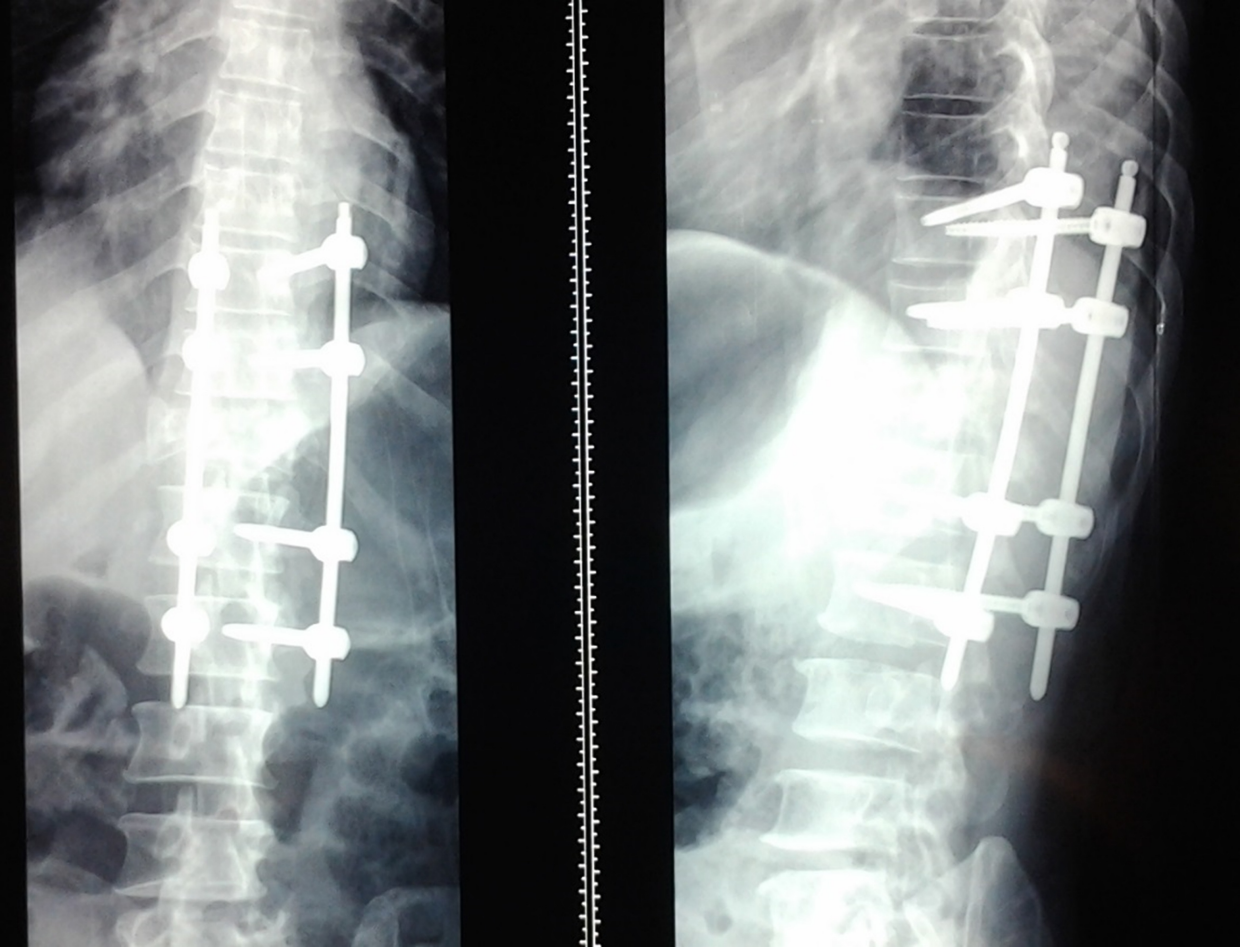
Postoperative Follow-up AP (left) and lateral (right) x-ray taken two weeks postoperatively demonstrating bridging instrumentation.

Using multiple small transverse fascial incisions resulted in minimal muscle trauma. The fascial incisions were closed individually, and an epifascial drain was placed. The skin was carefully tacked down to the muscle fascia in order to minimize dead space and avoid postoperative fluid collections. Postoperative x-rays demonstrated good placement of the instrumentation (Figure 10). The patient remained neurologically stable and was discharged home two weeks after surgery without postoperative complications. One year after surgery, the patient remains stable on neurological exam, but did not show signs of neurological improvement at that time. 

## Discussion

In large parts of Africa, including Tanzania, spinal trauma is routinely treated non-operatively with prolonged bedrest. Surgical treatment of unstable spinal fractures is the standard of care in more developed parts of the world. This is because it allows for decompression of neural elements and stabilization of the spine with early mobilization, which minimizes complications such as pulmonary emboli, infections, decubiti ulcers, etc. Obstacles to surgical treatment in Eastern Africa include the costs associated with instrumentation surgery, the lack of surgical training, equipment, and concerns regarding possible postoperative complications such as infection, wound breakdown, and need of revision surgery.

This case was performed as part of an ongoing initiative by Weill Cornell, the Foundation for International Education in Neurological Surgery (FIENS), and other organizations to improve neurotrauma care in East Africa [9-10].

### Limitations

We are fully aware of the difficulties and risks associated with the introduction of such technology in an underdeveloped country. For a country like Tanzania, the costs are currently still prohibitive, and local surgeons have to become more familiar with spinal surgery in general before adopting complex, minimally invasive and navigational technology.

These concerns notwithstanding, we believe that minimally invasive spinal surgery and navigation offers important benefits in resource-poor and underdeveloped regions. For a newer generation of spine surgeons, these technologies could become a routine part of their training. By performing the first MIS instrumentation and decompression procedures with 2D navigation together with our Tanzanian partners, we have shown promising opportunities. Spinal surgery in evolving nations will not have to duplicate the slow and laborious development of surgical techniques witnessed in Europe or North America. Rather, we believe that newer and less invasive techniques will be introduced at a faster rate with exposures to cutting-edge technology. This is currently being seen in the imaging sector in East Africa, as private radiological centers are advancing at a fast rate and offer access to high-quality imaging, such as MRI and CT.

## Conclusions

In summary, we have demonstrated the technical feasibility of introducing advanced, minimally invasive and navigation techniques to a university hospital setting in Tanzania. The introduction of spinal navigation with minimally invasive spinal surgery offers potential benefits, such as reduced surgical invasiveness, less blood loss, early patient mobilization, faster recovery, and lower wound breakdown and infection rate. With proper training, it has the potential to shorten the learning curve for spinal surgery and facilitate complex spinal procedures.
