# Correction: Minimally Invasive 2D Navigation-Assisted Treatment of Thoracolumbar Spinal Fractures in East Africa: A Case Report

**DOI:** 10.7759/cureus.c2

**Published:** 2016-06-06

**Authors:** Innocent Njoku, Othman Wanin, Anthony Assey, Hamisi Shabani, Japhet G Ngerageza, Connor D Berlin, Roger Härtl

**Affiliations:** 1 Department of Neurosurgery, Weill Cornell Medical College, New York Presbyterian Hospital, New York; 2 Department of Orthopedics, Muhimbili Orthopedic Institute (MOI), Dar es Salaam, Tanzania; 3 Department of Neurosurgery, Muhimbili Orthopedic Institute (MOI), Dar es Salaam, Tanzania

This article erroneously states that the main Brainlab office is located in Washington, IL. The correct location is actually Feldkirchen, Germany.

The relevant passage in the Introduction (third paragraph, second sentence) should read as follows: "The 2D Kick® system (BrainLab AG, Feldkirchen, Germany) is a relatively inexpensive navigational system (compared to 3D navigation) that combines the benefits of navigation with affordable purchasing and maintenance costs; therefore, its feasibility and use can be translated to assist surgeries in resource-limited regions of the world."

 

